# Simultaneous quantification of rDNA methylation and copy number: Constraints to natural variation in humans and cell lines

**DOI:** 10.1371/journal.pone.0336141

**Published:** 2025-11-13

**Authors:** Stephen Flint Smith, Bernardo Lemos

**Affiliations:** 1 Department of Pharmacology and Toxicology, R. Ken Coit College of Pharmacy, University of Arizona, Tucson, Arizona, United States of America; 2 Coit Center for Longevity and Neurotherapeutics, University of Arizona, Tucson, Arizona, United States of America; 3 Department of Environmental Health, Harvard T.H. Chan School of Public Health, Boston, Massachusetts, United States of America; University of the Punjab Quaid-i-Azam Campus: University of the Punjab, PAKISTAN

## Abstract

Ribosomal DNA (rDNA) copy number (CN) varies widely both across and within species. In humans, it has been hypothesized that rDNA transcriptional activity is partially regulated by rDNA methylation status. Here we describe a rapid and scalable approach for the simultaneous detection of methylated and unmethylated rDNA copy number using a well characterized digital PCR technology. The linear range for detecting rDNA CN and methylation is from 0.3 ng to 20 ng DNA with a practical range from 1.25 to 15 ng DNA and can be normalized with a single copy gene, ACTB, to reflect copies per genome. Our approach detects 28S rDNA methylation using a modified methyl-specific/bisulfite PCR targeting a region with at least four CpGs. In a population of healthy donors, we observed wide variation in both methylated and unmethylated rDNA CN, as well as in the percentage of methylated rDNA copies. We found significant correlations between the total rDNA CN and both methylated rDNA CN and percent methylated rDNA CN. Individuals with a greater number of rDNA copies had more methylated copies and a higher percentage of methylated copies. The methylated rDNA CN percentage varied widely across individuals and ranged from 9% to 69% of the total rDNA copies being methylated. Finally, four human cell lines (BEAS-2B, HeLa, MCF-7, MDA-MB-231) also displayed wide differences in methylated and unmethylated rDNA CN. Overall, this method provides a new tool to study the relationship between rDNA CN and methylation while offering insight into rDNA methylation variability and the regulation of active rDNA copies.

## Introduction

Ribosomal DNA (rDNA) consists of two genetic components: the 5S ribosomal RNA (rRNA) gene and the 45S rRNA gene. The 45S gene is localized on the short arms of the acrocentric chromosomes 13, 14, 15, 21, and 22, and is transcribed by RNA polymerase I [1]. The 5S rDNA is located on chromosome 1 and is transcribed by RNA polymerase III [2]. The 45S rRNA is transcribed as a single transcript and is post-transcriptionally modified to form the individual rRNAs 18S, 5.8S, and 28S which are essential for ribosome biogenesis [3]. rRNA transcription accounts for approximately 80% of all cellular RNA production and represents a significant metabolic investment [4,5]. The rDNA forms a key part of the nucleolus, a non-membrane bound organelle that is the site of rRNA production and ribosome assembly [6].

Genetically, the rRNA gene array is highly conserved, with the gene fragment the most conserved and the intergenic spacer susceptible to significant variation which corresponds to vastly different rDNA array size between organisms [7]. This is dramatically illustrated when comparing the yeast rDNA repeat to human rDNA [5]. They differ in size from ~9.2kb in yeast to ~43kb in humans [8,9]. In yeast, the rDNA undergoes repetitive gene expansion and constriction, which sensitizes the yeast to DNA damaging agents and regulates aging and senescence [10]. rDNA recombination and instability are well documented in yeast, Drosophila and humans. In yeast, rDNA recombination induces the formation of extrachromosomal circles, which can cause rDNA instability and lead to a decrease in organism lifespan [11–13]. In Drosophila regulating RNA polymerase II transcription of rDNA modulates rDNA copy number (CN) magnification, a critical mechanism for rDNA CN protection from extrachromosomal rDNA loss [14,15]. Recent telomere to telomere sequencing has enabled estimation of recombination rates of rDNA and has shown that the rate is higher for rDNA than for other sections of the genome [16]. In humans, changes in rDNA copy number can occur rapidly; differences in rDNA array number have been documented between generations, likely due to high rearrangement frequency during meiosis, 10% for each cluster [17].

In humans, the 45S arrays are a series of tandem repeats of the gene with some evidence suggesting that the arrays are arranged primarily in a head-to-tail pattern and some suggesting that the frequency of palindrome repeats, partial insertions, and inversions is fairly high [7,18,19]. Each chromosome has distinct rDNA sequence variation suggesting that intra-chromosomal recombination is the primary source of rDNA amplification [7]. A single 45S repeat contains the transcribed pre-rRNA and an intergenic spacer. The intergenic spacer is frequently methylated throughout and is consequently chromatin bound [19]. The intergenic spacer also houses several types of repeats that make it susceptible to some forms of recombination [19–22]. The repetitive nature of the rDNA gene repeats and the internal repeats support the hypothesis that rDNA is a hallmark of genome stability. The transcribed portion of rDNA is also methylated, in a gene-silencing dependent manner. Recent evidence suggests that methylation throughout the gene body, not just the promoter, denotes transcriptionally inactive regions [19,23–25].

Epigenetic regulation of rDNA is complex and is not necessarily consistent between various model organisms and humans. Drosophila, for instance, lacks methylation in the rDNA, whereas in humans DNA methylation seems critical for rDNA regulation, though the nuances of methylation at specific sites and regions remains poorly understood. A key gap remains in our understanding of rDNA methylation across its components (e.g., promoter regions vs gene body vs the IGS region). In transcriptionally active protein-coding genes, the promoter is typically unmethylated and the gene body is typically methylated [26,27]. However, extending this simple model to describe the rDNA has remained challenging. For the rDNA, one model suggests that some of the units are unmethylated from the promoter to the gene body while those that are not transcribed are methylated across that entire region [19,28]. Furthermore, it has been suggested that clusters of rDNA units could behave similarly. In one model clusters of ~20 consecutive repeats might all be transcriptionally active or might all be transcriptionally inactive [19]. It is hypothesized that these transcriptionally inactive clusters are formed into heterochromatin. In any circumstance, the mechanisms of rDNA epigenetic control and the role of DNA methylation across units and regions of the rDNA remains to be well characterized.

Within the human population, rDNA copy number is highly variable [7,29–31]. Estimates range from 50–250 copies per genome [29,31–33]. The reasons for such large variation in CN are poorly understood, but associations between CN and diseases indicate functional consequences [30,34–36]. It has also been suggested that environmental exposure or aberrant cell signaling can alter the copy number [30,37]. Further complicating understanding the causes of these CN changes and the downstream consequences of CN changes is the fact that only a fraction of the rDNA units is transcriptionally silent [24,32,38,39]. Compounding this issue, limited high resolution data is available regarding rDNA usage. Earlier estimates suggested that about 50% of the rDNA units were transcriptionally active, the remaining copies being silenced in a heterochromatic state [4,40–42]. Additionally, there is no satisfactory methodology to investigate a chromosome specific measure of rDNA copy number or methylation.

To understand the fraction of the rDNA array that is transcriptionally silent, we rely heavily on rDNA methylation detection, which riles on the hypothesis that silent heterochromatic units are heavily methylated [41]. Detection of methylated DNA relies primarily on bisulfite conversion of genomic DNA followed by sequence detection. Bisulfite conversion preferentially modifies unmethylated DNA cytosines and allows for nucleotide specific identification of methylation sites [43]. However, sequencing approaches (e.g., WGBS and RRBS) can be cost prohibitive, which limits their application. Methyl specific PCR (MSP) and its derivative methods were developed to measure the methylation status of targeted regions of the genome [44–46]. MSP relies on quantitative PCR of two distinct templates of DNA following bisulfite conversion [44]. We apply a modification of MSP to the simultaneous assessment of methylated and unmethylated rDNA copy numbers. Our data suggest that the number of unmethylated (active) rDNA CN can be rather low, and that the fraction of methylated rDNA copies can be highly varied. Furthermore, the data suggest that neither the total rDNA CN nor the percentage methylated rDNA copies correlates with age. This proposed method provides a scalable high throughput approach to assess both rDNA CN and methylation percentage.

## Results

### Digital methylated copy number assessment

We developed a dPCR methodology, called digital methylated copy number (dMCN), which estimates methylated and unmethylated rDNA copy numbers. It also estimates the fraction of methylated rDNA units relative to the total number of rDNA units (% rDNA methylation). These measurements provide a simple high throughput method to investigate the relationships between total rDNA CN (tCN), methylated CN (mCN), unmethylated CN (uCN), and percent methylation.

**Fig 1:**
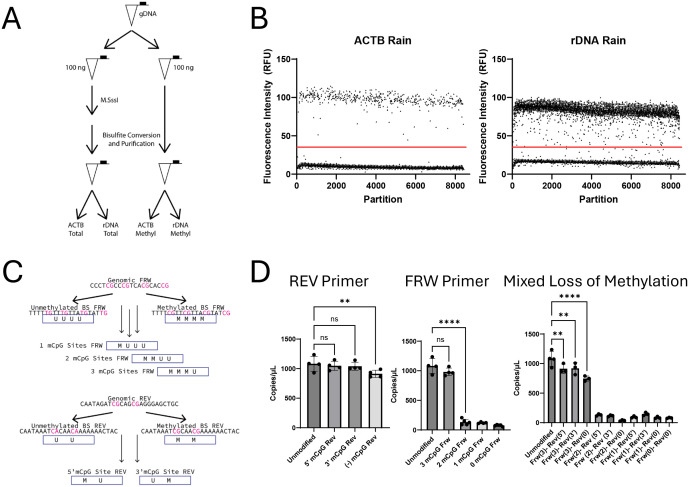
Methylation detection in the rDNA 28S gene body by MSP is robust and sensitive to methylation state of specific sites. **A** Scheme of dMCN protocol. A single sample of gDNA was normalized to 10 ng/µL and then 100 ng aliquots were individually bisulfite converted or methylated with M.SssI followed by bisulfite conversion. Both aliquots were then measured for ACTB and methylated rDNA. **B** Representative partition fluorescence plots show the positive and negative populations and the “rain” for each PCR reaction. **C** Scheme describing the design of primers used for methylation specific testing. Primers were designed to be complementary to bisulfite converted DNA with unmethylated CpGs. **D** Loss of methylation results in a decrease in number of copies detected. For the reverse primer methylation at one position yields equivalent detection. For the forward primer methylation at 3 positions yields equivalent detection. With mixed loss of methylation, 4 total sites yields near equivalent detection (n ≥ 3 Ordinary One way ANOVA adjusted P value, ** p-value < 0.01, **** < 0.0001).

Traditional MSP uses two primer pairs, one to detect methylated template and one to detect unmethylated DNA [44]. This approach requires optimizing a duplex qPCR reaction with bisulfite converted DNA, which is technically challenging and limits the genomic sequences that can be used. Bisulfite conversion reduces DNA heterogeneity, which alters the annealing temperature range for unmethylated versus methylated templates and complicates PCR optimization. dMCN simplifies some of these technical challenges by measuring only methylated DNA. Briefly, a single sample of genomic DNA (gDNA) is divided into two equal aliquots. One aliquot is treated with the methyltransferase M.SssI to methylate all CpG sites, referred to as Total, and one aliquot is not treated, referred to as Methylated, as shown in Fig 1A. After *in vitro* methylation, each aliquot is bisulfite-converted and analyzed by dPCR to measure methylated rDNA CN (tCN or mCN). dMCN uses ACTB as both an internal standard and a single-copy reference to control for bisulfite conversion and purification efficiency [47]. The primers used for ACTB amplification do not span a CpG site and are thus insensitive to M.SssI treatment. Because the amplicon target for ACTB is present in equal amounts when the aliquots were taken and is insensitive to M.SssI treatment, it should yield identical measurements for both Total and Methylated aliquots. Indeed, we observed strong agreement between ACTB copies/µL for each paired aliquots and consistent ranges between batches (S1 Fig.).

We designed methyl specific amplicons within the rDNA gene to find representative sites that would reflect the methylation status of the rDNA. Shown in S1B Fig. are the locations within the gene of sites previously used for rDNA CN estimation and some of the new amplicons that we investigated for dMCN [48]. After testing the amplification of these targets with bisulfite converted template, we chose a target within the 28S portion of the gene that includes a total of 6 CpG sites within the primers. One criterion for selection of viable amplicons was the minimization of “rain” during dPCR [49,50]. The partition plots of ACTB and rDNA (Fig 1B) show representative plots of the dPCR results from these two reactions.

### dMCN-PCR specificity

For dMCN, we used a forward primer (FRW) with four CpG sites and a reverse primer (REV) with two CpG sites (Fig 1C). To assess the sensitivity of our primers to methylation of the rDNA, we fully methylated gDNA with M.SssI treatment and measured the number of copies/µL for a single reference sample. We then measured the copies/µL of this reference sample using primer combinations designed to match different methylation states at each site. Fig 1C illustrates the rationale of the primer combinations to mimic loss of methylation in the CpG sites.

For the REV primer, we found that dMCN was unaffected by the loss of either methylation site individually; however, the absence of both methylation sites resulted in a slight decrease in the measured copies/µL (compare 5’ CpG/3’CpG and (-) CpG in Fig 1D). When no methylated CpGs are present in the REV primer, (-) CpG, we observed a statistically significant decrease in estimated copies/µL compared to fully methylated primers; however, the copies/µL estimate remained elevated when compared to reactions where both primers are unmethylated. When we compared the partition plot for: fully methylated primers, unmethylated REV, and unmethylated primers (S2 Fig.), we still observed a sizable population of positive partitions with the (-) CpG REV primer and a fully methylated FRW primer. This suggests that for a mixed methylation template like a biological sample, if all the template molecules were unmethylated in the REV sites, there would still be a sizable number of positive partitions when the FRW sites are methylated.

When examining the FRW primer binding site we saw a similar response with primers designed for three methylation sites. We detected many positive partitions when using primers with three methylated CpGs (mCpGs) in the FRW primer and two in the reverse but saw a significant decrease in positive partitions when only using four total mCpGs (Fig 1D). This suggests that dMCN requires at least 3 of the 4 sites in the FRW primer to be methylated for suitable dPCR detection. For the FRW primer, the loss of more than one methylated site led to a greater reduction in the number of positive partitions than any loss observed with the REV primer combinations.

**Fig 2:**
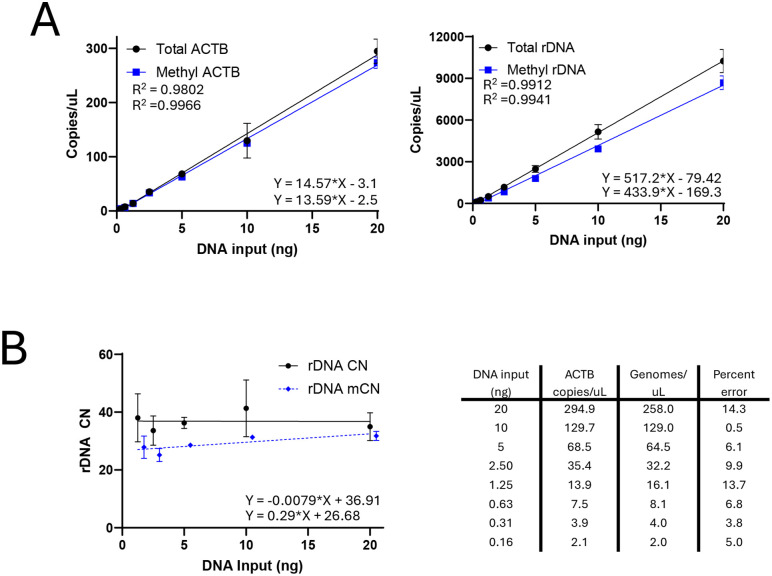
Methylation specific PCR is reproducible within a wide dynamic range. **A** Both detection of a single copy gene and rDNA are linear between 0.3 ng and 20 ng per reaction. The single copy gene ACTB is insensitive to methylation status and detects the same copies/µL with and without M.SssI treatment. Methylated rDNA has lower copies/µL than total rDNA as expected. DNA input is for a 12 µL dPCR reaction with the number of copies per µL indicating the absolute quantification from dPCR. **B** When normalized to ACTB, rDNA tCN and mCN are stable across our detection range with mCN consistently less than tCN. rDNA CN reflects normalization to ACTB independently for native (mCN) and M.SssI treated (tCN) aliquots. When compared to theoretical genome copies/ µL we report < 15% error consistently with an average percent error of 7.5%.

We then tested the loss of methylation in both FRW and REV primers in combination and observed effective detection of methylated DNA only happens when at least four of the six sites were methylated. The partial recognition of unmethylated sites impacted our quantification of rDNA methylation and likely manifests in the greater “rain” we observed for our rDNA methylation compared to ACTB which is insensitive to methylation. However, this artifact would bias results towards overestimating total mCpGs and depends on the ratio of methylated to unmethylated CpGs within the primer recognition sequence, which may actually enhance our representation of global rDNA percent methylation. Not only did we observe more “rain” in our dMCN but when testing primers with fewer mCpGs, the “rain” for those reactions was elevated, and there was often a lack of a distinct population of positive partitions in the raw dPCR data (S2 Fig 2). Combined, these data suggest that dMCN detects a methylated rDNA copy when there are at least 4 mCpGs in our targeted sites and represents a measurement of the global strand methylation percentage.

### rDNA copy number and methylation detection

We observed a linear response for both ACTB and rDNA dPCR in serially diluted Total (M.SssI-treated) and Methylated (native) samples. We also observed strong reproducibility across DNA inputs ranging from 0.2 ng to 20 ng for all individual dPCR reactions, with consistent results observed in both technical replicates and independent assay runs (Fig 2A). gDNA input above 20 ng caused positive partition saturation and prevented accurate dPCR quantification on the QIAcuity 8.5K plate but also neared the upper limit of detection even with additional partitions. Conversely, while we can reproducibly quantitate single copy genes with low DNA input, this resulted in so few positive partitions that it is below the practical usage limit and increases the coefficient of variation. During subsequent testing of variable human data, we found that 10 ng DNA input allowed for consistent quantification of ACTB, and 1 ng DNA input for rDNA allowed enough dynamic range to cover the wide range of rDNA CN.

We then measured the consistency of rDNA CN estimation in our serial dilution series. Normalization to ACTB yielded consistent copy number estimates per genome across the gDNA input range of 1.25 to 20 ng (Fig 2B). As expected, mCN values were consistently lower than tCN. We also compared genome estimates derived from ACTB to theoretical values based on input DNA mass. Our test DNA came from BEAS-2B cells, which originated from a male donor with ~6.41 pg per genome, which was used to calculate the theoretical input genomes per µL. Because bisulfite-converted DNA was used, only one copy of ACTB per genome is expected instead of two. Across the DNA input range, we report a percent error of <15% with the lowest error in the 10 ng input DNA reaction with less than 1% error. These data together suggest that dPCR measurement of single copy, multi copy, and methylation status is reliable and can be ascertained across a broad dynamic range.

### Copy number of methylated and unmethylated rDNA is highly variable across individuals and immortalized cell lines

**Fig 3:**
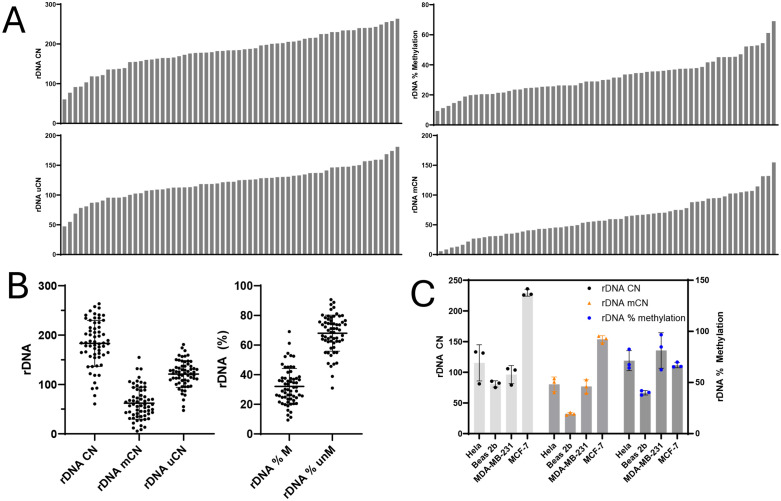
Inter-individual variability in total rDNA CN, unmethylated rDNA CN, methylated rDNA CN, contributes to similarly wide variability in percent of methylated rDNA copies. **A,B** rDNA tCN range from 60.49 to 263.7 copies per haploid (equivalent to between121 and 527.4 copies per cell). rDNA uCN range from 47.39 to 180.9 copies per haploid. rDNA mCN range from 5.58 to 154.8 copies per haploid. The methylation percentage was from 9.3% to 69%. The average tCN, uCN, mCN, and percent methylated were 183.2, 121.1, 62.05, and 32.01% respectively. (n = 62) **C** rDNA CN is variable between commonly used model cell lines and the tCN does not consistently predict rDNA percent methylation, specifically for MCF-7. All estimates are CN per haploid genome (n = 3).

To investigate variation in methylated and unmethylated rDNA copy number in humans, we analyzed blood derived DNA from a population of 62 individuals. The average age of our population was 59.5 years and ranged from 18 to 88 years. Males represented 45.2% of our population. For this population, we observed a total rDNA CN that ranged between 61 and 264 copies per haploid genome, with a mean of 183 copies (Fig 3A, B). The rDNA uCN ranged between 47 and 181 copies with an average of 121 unmethylated rDNA copies per haploid genome. The rDNA mCN ranged between 6 and 155 copies with a mean of 62 methylated rDNA copies per haploid genome. Finally, the average percent methylation of rDNA ranged between 9.33% to 69.03% with an average of 32% methylation per genome (Fig 3A, B). Percent methylation refers to the percentage of rDNA copies that are methylated in the coding region, specifically measured by six CpG sites. Our estimate of an average percent methylated agrees with previously reported sequencing analyses of global rDNA methylation [19,33,51–53].

We also measured the rDNA CN and methylation for four immortalized cell lines. Three of our cell lines (MCF-7, MDA-MB-231, and HeLa) were derived from breast and cervical cancers, whereas BEAS-2B are lung epithelial cells immortalized with adenovirus 12-SV40 hybrid virus. HeLa, MDA-MB-231, and BEAS-2B showed similar rDNA tCN, which was surprising, as we would have expected the three cancers to be more similar (Fig 3C). We observed that MCF-7 cells showed the highest rDNA CN while having a similar methylation percentage to the other cancerous cells. BEAS-2B, the one non-cancerous cell line, presented with significantly lower rDNA percent methylation. BEAS-2B cells are near diploid while the other cells are aneuploid and have significant chromosome duplication [54–60]. Ploidy changes are expected to affect our estimate of rDNA CN due to the assumption that the ACTB gene is present as a single copy gene. This is also true for the rDNA in case of aneuploidies of the 5 chromosomes containing the rDNA. Hence, it is possible that ploidy changes (in the rDNA or the ACTB gene) might partially explain why MCF-7 has significantly greater rDNA CN compared to the other cell lines. These results, in conjunction with the high variability in rDNA tCN and methylation percentages observed in our human population, underscore that the number of methylated copies is only partially explained by tCN.

### Relationship between rDNA uCN, mCN, and tCN

**Fig 4:**
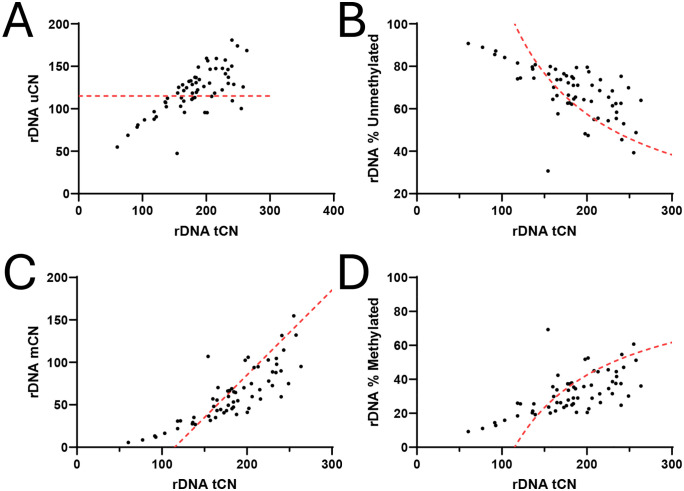
Total rDNA CN (tCN) correlates with unmethylated rDNA CN (uCN), methylated rDNA CN (mCN), and percent of methylated and unmethylated rDNA copies. **A-D** rDNA tCN correlates with uCN, mCN, % unmethylated, and % methylated. (Spearman correlations r = 0.69, 0.83, −0.66 and 0.66, respectively, n = 62, p < 0.0001 for all). The red dashed line represents the Hori et al (2021) hypothesis that there is a constant number of 115 active rDNA units per haploid genome (uCN). Note that (i) some individuals have fewer than 115 total rDNA CN (tCN), and that (ii) the number of unmethylated rDNA (uCN) is not consistent across individuals and is rarely more than 85% of the total rDNA copies.

Next, we assessed the relationships among methylated (mCN) copy number, unmethylated (uCN) rDNA copy number, and total rDNA copy number (tCN). We observed strong positive correlations between rDNA tCN and mCN, uCN, and methylation percentages (Fig 4A-D). We represented the Hori et al (2021) hypothesis of steady state active rDNA CN with a red dashed line in Fig. 4. Note that (i) some individuals have fewer than 115 total rDNA CN (tCN), and that (ii) the number of unmethylated rDNA (uCN) is not consistent across individuals and is rarely more than 85% of the total rDNA copies. Our data was in agreement with previously reported estimates with bisulfite and nanopore sequencing that suggested rDNA percent methylation correlates with rDNA CN [19,33,51]. The correlation suggests that the fraction of the active rDNA is partially determined by the total rDNA units available: individuals with a larger number of rDNA units present with a greater number of methylated copies. Highly methylated units are presumably inactive.

**Fig 5:**
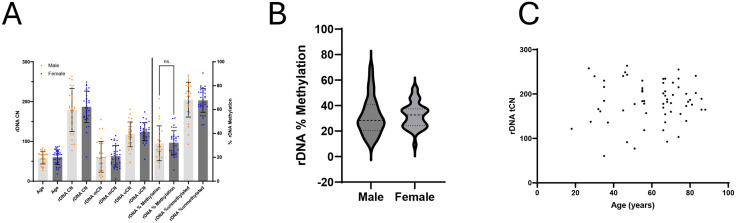
Percent methylation is more disperse in males. **A** Male patients have greater variance in % methylation than do female patients. (Comparisons were made using Wech’s T test, F test p = 0.0395, Male n = 28, Female n = 34) **B** Density histogram of percent methylation based on sex shows the increased variance of male samples. **C** There is no correlation between rDNA tCN and age. (r = −0.01344, Spearman’s correlation, p = 0.9174).

When comparing our representative population by sex, we observed that there was no difference in the mean or median of our measurements, but we noted that the male population tended to have a greater spread compared to the female population (Fig 5A). When analyzed with an F-test, only the percentage methylation showed a statistical difference in spread (Fig 5B). rDNA methylation and CN have also been associated with age, but recent reports have not seen this same correlation; subsequently, we sought to distinguish if there is a correlation in our data [19,61,62]. We did not observe any correlation between age and tCN, mCN, uCN, or methylation percentage (Fig 5C).

## Discussion

In order to meet the extraordinarily high demand for rRNAs the rDNA is typically present in high copy numbers in organisms as diverse as yeast, fruit flies, worms, and humans. As the genetic template for rRNA, an essential component of ribosomes, rDNA drives ribosome biogenesis and is essential for cell survival, requiring the highest transcriptional activity of any gene to meet the demand for rRNA production. In humans, the copy number of the rDNA can vary over a 10-fold range, it has been found as low as 61 copies per individual but also as high as 1590 in some individuals [31]. However, the relationships between rDNA copy number and rDNA methylation has remained uncertain [36,40,63–65]. Although a minimum threshold of transcriptionally active rDNA copies is thought to be necessary to supply the minimum rRNAs needed for cell survival, the homeostatic balance between unmethylated (transcriptionally active) and methylated (transcriptionally inactive) copies remains unresolved. We hypothesized that rDNA copy number and methylation is constrained within a biologically permissive window to match cellular requirements for rRNA components of the ribosome. This window might not only be bounded by a minimum number of active rDNA units required for viability, but also bounded by an upper limit beyond which excess rRNA transcription might become detrimental [4,66–68]. We expect that an upper limit could emerge from high costs of rRNA production if rDNA transcription exceeds the cellular needs for ribosome biogenesis. Excessive rRNA production has also been shown to create other toxic side effects for the cell [4]. Accordingly, here we observed a correlation between total rDNA CN and both unmethylated and methylated copy number. Interestingly, the variability in the percentage of methylated copies increases as the total rDNA copy number increases. For instance, the six individuals with the smallest rDNA copy number had fewer than 120 total copies and about 15–20% of them methylated. In contrast, the six individuals with the highest rDNA copy numbers, each exceeding 230 copies, had between 25% and 62% of their copies methylated. Furthermore, unmethylated copies rarely exceed 85% of total rDNA. (Fig 4A). The corollary observation is that methylated CN approaches a slope of 0.15, again suggesting that methylated CN cannot be less than 15% of the total CN (Fig 4C). This challenges two hypotheses about rDNA copy number regulation: first, that a relatively high minimum number of active rDNA copies is required for cellular function; and second, that as total rDNA copy number decreases, the percentage of active copies to total copies increases to compensate.

We further wanted to compare our estimation of rDNA methylation with that of previously published work using nanopore sequencing. *Hori et al.* (2021) reported a total rDNA CN ranged between 250–700 copies per cell across 23 individuals. This is in line with prior suggestions from short read sequencing indicating a 10 fold variation in rDNA copy number [17,29,31,51]. *Hori et al* also observed a negative correlation between the ratio of uCN/tCN and tCN. We observe this negative correlation between tCN and percent unmethylated as well (Fig 4B). *Hori et al.* also supported the hypothesis that the active number of rDNA copies in a cell (i.e., unmethylated CN) is 230 copies per cell (i.e., 115 copies per haploid genome), regardless of the individual total CN [19]. Here we represent that hypothesis as the red dashed lines in Fig 4. Our data suggests that the number of active unmethylated rDNA can instead be quite variable, ranging from about 50–180 unmethylated copies per haploid genome.

It is noteworthy that methylation is only one of the mechanisms regulating rDNA transcription, rDNA organization, and ribosome biogenesis. Additional regulatory pathways and epigenetic modifications also contribute. This is most evident in the case of fruit-flies (Drosophila), an organism that lacks DNA methylation-based regulation of rDNA activity [69,70]. Our study is also limited in scope due to using bulk DNA sampling from both cell culture and DNA from blood. Bulk blood DNA represents a heterogeneous population of cells and thus cannot be used to infer cell specific events. However, this type of DNA has been used widely for diagnostic and investigational population genetics studies. Furthermore, our study relied on bisulfite conversion. While a well-established method, bisulfite conversion can induce artifacts due to variation in the bisulfite conversion efficiency. Even though we did not fully characterize the bisulfite conversion efficiency, we included as multipurpose control, ACTB, which helps normalize bisulfite induced artifacts. We also used PCR quantitation which can induce bias, particularly when using the simplified template from bisulfite conversion. However, the primers we used for ACTB quantitation are widely used for methyl specific PCR and perform robustly across sample type and PCR techniques.

Together, our findings support a model in which rDNA transcriptional activity is partially determined by rDNA copy number and is regulated within a constrained window, rather than fixed at a universal threshold across individuals. While prior studies have proposed a constant number of active rDNA copies per cell, our data demonstrate considerable inter-individual variability in unmethylated rDNA copy number, suggesting that the balance between active and inactive rDNA is dynamically adjusted. That unmethylated rDNA rarely exceeds ~85% of the total, irrespective of copy number, suggests that the upper limit of rDNA activity could also be constrained by features of genomic architecture and rDNA organization rather than by metabolic requirements. These regulatory constraints may reflect both an energetic burden of excessive rRNA synthesis as well as a need to preserve genomic stability and should be investigated further. While our study and others infer transcriptional activity of rDNA based on rDNA gene and promotor methylation, few studies have linked measures of rRNA transcription and processing with either rDNA CN or methylation status. In a Cas9 targeted methylation study, Blokhina and Buchwalter, reported that inducing hypermethylation did not affect rRNA transcription, but further study is warranted [28]. Altogether, these findings highlight the importance of dosage-sensitive regulation of rDNA activity in human cells.

## Materials and methods

### Cell culture and DNA extraction

HeLa, MCF-7, MDA-MB-231, and BEAS 2B were cultured in DMEM with 4.5 g/L glucose, L-glutamine and sodium pyruvate (Corning 10–013-CV) supplemented with 10% FBS (Corning 35–011-CV) and 1% Penicillin/ Streptomycin/ Amphotericin B solution (Corning 30–004-CI). Unless specified otherwise, DNA was extracted from cells that had undergone fewer than 15 passages since their acquisition from ATCC. DNA was extracted from cells with Qiagen DNeasy Blood and Tissue Kits following the manufacturer protocol with minor modifications to elute in 75 µL of MQ water for both first and second elutions instead of the recommended volume and buffer. Samples were quantified by Qubit Flex with dsDNA BR assay kit following the manufacturer protocol (ThermoFisher). DNA was stored at – 20 °C.

### M.SssI CpG methylation

Purified DNA was methylated with M.SssI from NEB (M0226L) and used as directed. Briefly, 100 ng of DNA(10 µL) was combined with NEB buffer 2, SAM (400 µM), M.SssI (4 units), and water followed by incubation at 37 °C for 1 h and then 65 °C for 20 min. DNA was subsequently stored at 4 °C or bisulfite converted immediately.

### Bisulfite conversion

Two bisulfite conversion methods were employed depending on scale of DNA to produce. When preparing large amounts of DNA for measuring a standard curve of our rDNA target and detecting methylation specificity, we used EZ DNA methylation Lightening kits from Zymo Research (ZD5031) and used 750 ng DNA per reaction eluted in 18.75 µL. When measuring DNA of limited quantity or for targeted measurement, we used the same kit with 100 ng of DNA as input and eluted in 20 µL.

To convert the DNA we followed the manufacturer protocol. Briefly, 130 µL of Conversion reagent was added to 20 µL of DNA (100 ng) and incubated at 98 °C for 8 minutes followed by 54 °C for 1 h. M-binding buffer was mixed with the reaction and applied to a DNA purification column. Following loading the column and washing once with M-Wash buffer. After washing once, 200 µL of L-desulphonation buffer was incubated with the column at room temperature for 20 minutes followed with two more washes and then elution of DNA in 20 µL of MQ water. We did not verify conversion efficiency but instead relied on conversion efficiency to be represented by variance of ACTB detection.

### Methyl specific dPCR

Because methyl specific dPCR uses bisulfite converted DNA as template, which is fragmented, we did not enzymatically digest the gDNA prior to dPCR. We used a Qiagen QiAcuity dPCR system to conduct the methyl specific PCR. For each reaction we used Qiagen EG PCR master mix (250112) and prepared reactions according to the manufacture instructions. We used QiAcuity 8.5K plates both 24 well and 96 well to conduct dPCR and we used auto thresholding to detect positive and negative partitions. Briefly we combined 4 µL of EG MM with 2 µL of respective template, 4.8 µL of water, and 1.2 µL of 4 µM primer mixes with both forward and reverse primers. PCR reaction conditions were as follows: Initial denaturation 95 °C for 5 minutes, 95 °C for 30 sec, annealing temperature (ACTB-58°C, M2- 50°C) for 30 sec, 72 °C for 30 sec. These temperatures were cycled 40 times and followed by a 5-minute incubation at 40 °C prior to image collection. Primers are listed in S1 Table.

### Methyl specific PCR linear range determination

Four bisulfite conversion replicates were conducted on the same sample of DNA extracted from Beas 2B cells to provide technical replication of the combined conversion and PCR methodology. Due to the amount of DNA required for the number of dilutions planned, each replicate consisted of 4 aliquots of 750 ng bisulfite conversions that were pooled prior to dilution. DNA was eluted in 18.75 µL of water per 750 ng to yield DNA nominally at 40 ng/µL. Samples were serially diluted from 10 ng/µL to 0.078 ng/µL by diluting in half for each step. These dilution series were created for both total (M.SssI treated) and methylated DNA samples. These samples were then measured in triplicate across 4 96 well dPCR plates: Plate 1-Total ACTB, Plate 2-Methylated ACTB, Plate 3-Total M2 rDNA, and Plate 4-Methylated M2 rDNA. Please see the data for replicates in S2 Table. Following averaging of dPCR triplicate, the bisulfite conversion replicates were analyzed with linear regression.

### Primer methylation specificity

To measure the specificity of our primers for methylated DNA, we designed a series of primers complementary to bisulfite converted template without methylation and intermediate levels of methylation. We then generated a reference template by treating gDNA with M.SssI as previously described and bisulfite converting 600ng of Beas-2B gDNA. The primer pairs from full methylation to zero methylation were then used to measure the copies/ µL of this reference sample.

### Human DNA samples

DNA samples from human blood donations were procured from commercially available healthy donor samples sourced from Reprocell and the sample collection and consent were approved by their appropriate IRB. The reference samples for the biosamples are included in S3 Table. We used 62 samples aged between 18–88 years old with 28 male and 34 female origin samples. Please see S3 Table.

## Supporting information

S1 Fig Copies/µL of single copy reference gene ACTB between paired aliquots. **A** Paired aliquots show strong agreement for copies/ µL of ACTB in both aliquots. **B** Diagram of rDNA array. Black lines indicate PCR targets for traditional rDNA CN estimation and methyl specific PCR. M2 indicates relative position of primer pair used for dMCN.
(PDF)

S2 Fig Loss of methylation in the REV primer site still produces many positive partitions. When compared to unmethylated primers and methylated primers, REV primers without methylation produce a well separated positive population. This positive population contributes to the positive population of a mixed methylation template and is reflected in the quantification.
(PDF)

S1 Table Primers used in this study. (XLSX)

S2 Table Raw data for Serial dilution of DNA. (XLSX)

S3 Table List of human samples used and rDNA CN measurements. (XLSX)
