# Supplementary figures and images for: Simultaneous quantification of rDNA methylation and copy number: Constraints to natural variation in humans and cell lines
Source: PLoS One. 2025 Nov 13;20(11):e0336141. doi: 10.1371/journal.pone.0336141 (PMC12614545; doi:10.1371/journal.pone.0336141)

S1Fig

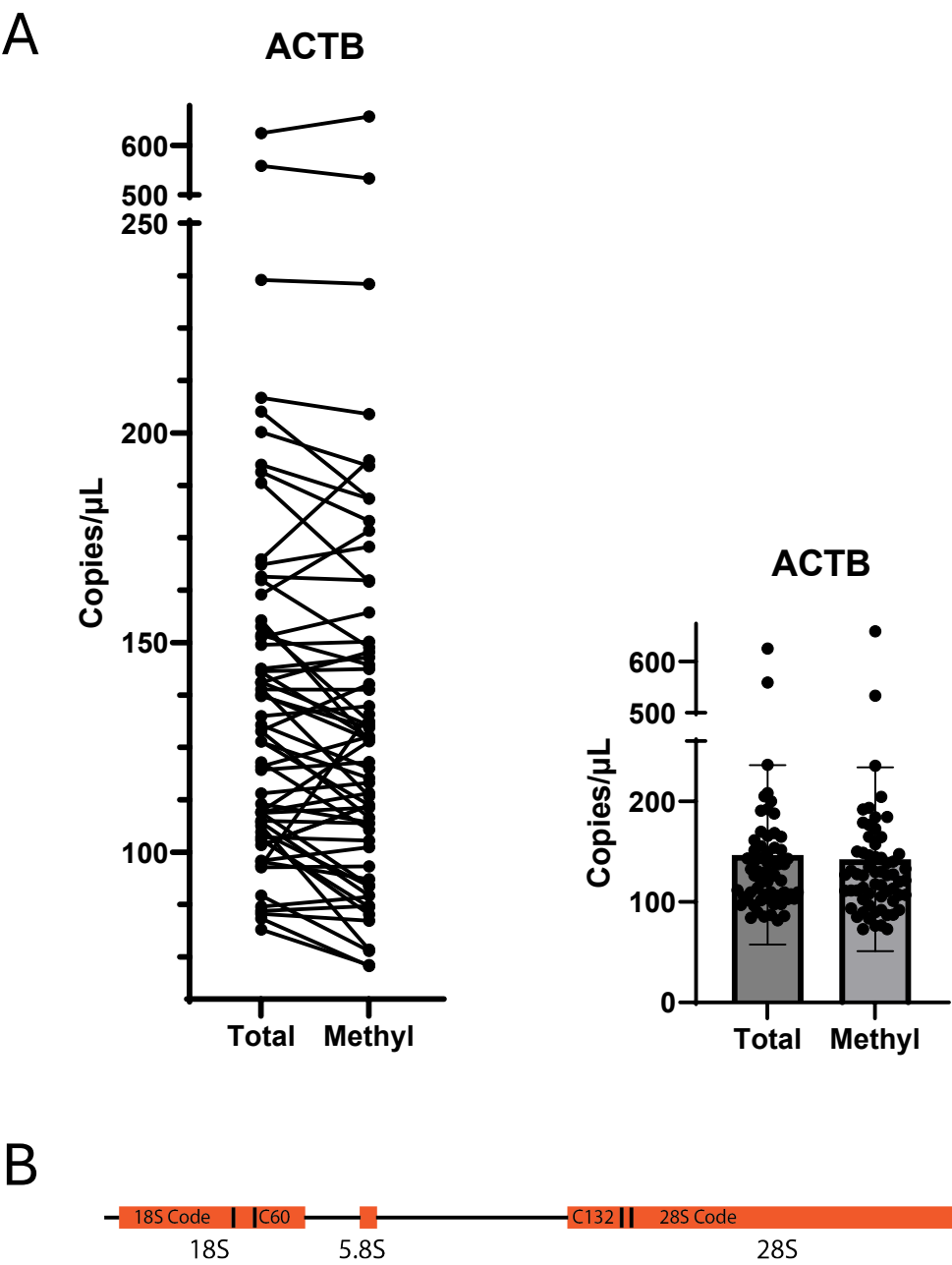

Supplement: S1 Fig — A Paired aliquots show strong agreement for copies/ µL of ACTB in both aliquots. B Diagram of rDNA array. Black lines indicate PCR targets for traditional rDNA CN estimation and methyl specific PCR. M2 indicates relative position of primer pair used for dMCN. (PDF) [file pone.0336141.s001.pdf]

S2 Fig

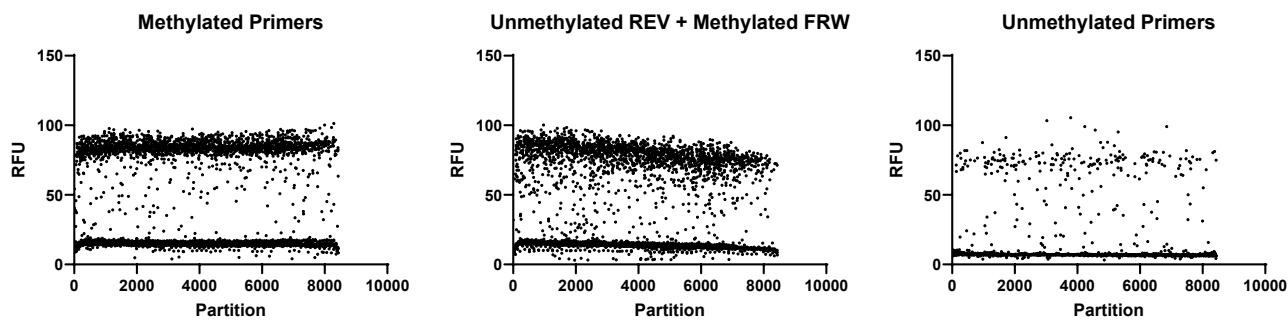

Supplement: S2 Fig — When compared to unmethylated primers and methylated primers, REV primers without methylation produce a well separated positive population. This positive population contributes to the positive population of a mixed methylation template and is reflected in the quantification. (PDF) [file pone.0336141.s002.pdf]
